# Emerging roles of the Hedgehog signalling pathway in inflammatory bowel disease

**DOI:** 10.1038/s41420-021-00679-7

**Published:** 2021-10-26

**Authors:** Zhuo Xie, Mudan Zhang, Gaoshi Zhou, Lihui Lin, Jing Han, Ying Wang, Li Li, Yao He, Zhirong Zeng, Minhu Chen, Shenghong Zhang

**Affiliations:** Division of Gastroenterology, The First Affiliated Hospital, Sun Yat-sen University, Guangzhou, P. R. China

**Keywords:** Inflammatory bowel disease, Cell signalling

## Abstract

The Hedgehog (Hh) signalling pathway plays a critical role in the growth and patterning during embryonic development and maintenance of adult tissue homeostasis. Emerging data indicate that Hh signalling is implicated in the pathogenesis of inflammatory bowel disease (IBD). Current therapeutic treatments for IBD require optimisation, and novel effective drugs are warranted. Targeting the Hh signalling pathway may pave the way for successful IBD treatment. In this review, we introduce the molecular mechanisms underlying the Hh signalling pathway and its role in maintaining intestinal homeostasis. Then, we present interactions between the Hh signalling and other pathways involved in IBD and colitis-associated colorectal cancer (CAC), such as the Wnt and nuclear factor-kappa B (NF-κB) pathways. Furthermore, we summarise the latest research on Hh signalling associated with the occurrence and progression of IBD and CAC. Finally, we discuss the future directions for research on the role of Hh signalling in IBD pathogenesis and provide viewpoints on novel treatment options for IBD by targeting Hh signalling. An in-depth understanding of the complex role of Hh signalling in IBD pathogenesis will contribute to the development of new effective therapies for IBD patients.

## Facts

- The Hh signalling pathway is implicated in maintaining intestinal homeostasis.
- The Hh signalling pathway interacts with multiple pathways that play critical roles in IBD.
- The role of Hh signalling in the pathogenesis of IBD and CAC remains ambiguous.

## Open questions

- What is the specific mechanism of Hh signalling in IBD pathogenesis?
- Does noncanonical Hh signalling affect the pathogenesis of IBD?
- Can we develop novel and potent drugs for IBD and CAC patients by targeting Hh signalling?

## Introduction

Inflammatory bowel disease (IBD), including ulcerative colitis (UC) and Crohn’s disease (CD), is a chronic and relapsing inflammatory disorder of the gastrointestinal tract [1]. As in Western countries, their incidence has been rising in newly industrialised countries since 1990 [2]. Thus, IBD has become a global disease and an enormous public health burden. Importantly, its incidence is high in young adults, the major labour force of society [3]. To prevent disease flares and progression, valid and long-term treatment is generally recommended at the disease onset in young patients. However, the pathogenic mechanisms of IBD are not fully understood, and effective IBD treatments are urgently needed. IBD pathogenesis is related to the convergence of environmental, microbial, immunological, and host genetic factors [4]. Hedgehog (Hh) signalling plays a critical role in intestinal homeostasis and is closely associated with intestinal inflammation and tissue repair [5]. Emerging data suggest that the Hh signalling pathway is strongly involved in IBD pathogenesis and may be a novel therapeutic target for IBD treatment [6]. In this review, we summarise the mechanisms and functions of Hh signalling, its role in IBD and colitis-associated colorectal cancer (CAC), and discuss the research directions required for the development of more effective IBD treatments.

## The Hh signalling pathway

*Hh* was initially identified in a genetic screen for segment-polarity in *Drosophila* [7]. This pathway is highly conserved [8, 9] and plays a critical role in homeostasis and multiple developmental processes, such as intestinal development and homeostasis [5]. The Hh family of intracellular signalling proteins is present in numerous invertebrate and vertebrate species [8, 10–12]. Genetic loss of *Hh* results in *Drosophila* larvae resembling hedgehogs, due to the spiky cuticle, hence the name Hedgehog. Mammalian Hh signalling comprises three secreted Hh protein ligands named Sonic hedgehog (Shh), Indian hedgehog (Ihh), and Desert hedgehog (Dhh) [8]. The main components of Hh signalling include the Patched receptors (Ptch1, Ptch2), the secondary membrane receptor Smoothened (Smo), inhibitor suppressor of fused homologue (Sufu), and Glioma-associated oncogenes (Gli1, Gli2, Gli3, homologues of *Drosophila* Cubitus interruptus (Ci)), which are downstream transcription factors [11].

### Canonical Hh signalling

Hh ligands interact with receptors and co-receptors on the cell membrane and trigger an intracellular cascade, thereby causing a change in target gene expression. In detail, Hh ligands bind to Ptch, initiating a complex signalling cascade that culminates in the activation of the transcription factor, Gli1, the downstream effector of the pathway. This signalling pathway is known as “canonical” Hh signalling.

Once Hh precursor proteins are produced, they are sent to the endoplasmic reticulum and Golgi body for protein processing and modification [13]. Cholesterol-modified and palmitoylated Hh proteins are anchored to the cell membrane due to their lipid modifications but are also secreted with the assistance of several factors [14], ensuring that local and distant signal transduction is tightly controlled. The membrane transporter Dispatched1 (Disp1), which is composed of a 12-pass transmembrane domain, is required for long-range signalling [15, 16]. Disp1 binds directly to the cholesterol moiety of human Shh ligands and thus enhances Shh membrane extraction [17]. Additionally, heparan sulphate proteoglycans promote Hh signalling by facilitating extracellular Hh transport [18]. On the cell membrane, Ptch and Smo are the core receptors that transmit the Hh signal [19]. Ptch encodes a 12-pass transmembrane protein and in mammals, two Ptch genes (Ptch1 and Ptch2) have been identified [20]. Both Ptch1 and Ptch2 recognise various Hh ligands (Shh, Ihh, and Dhh) with similar affinity, but the tissue distribution of these two receptors does not fully overlap [21]. Smo, a 7-pass transmembrane protein, is the activator of the downstream pathway and belongs to class F G protein-coupled receptors [20]. The primary cilium (PC), where multiple processes occur, is required for the response to Hh signalling in vertebrates, and the destruction of PC components attenuates the response to Hh ligands [22]. Corbit et al. [23] found that Smo localises to the PC in an Hh-dependent manner. The absence of Hh ligands leads to the inhibition of Smo activity by Ptch. In contrast, the binding of the Hh ligand to Ptch1 prevents inhibition of Smo, a receptor necessary for Hh signal transduction [24], which drives the accumulation and activation of Smo from the plasma membrane to the PC [14], thereby initiating a complex signalling cascade that leads to the activation of Gli transcription factors. Cholesterol promotes Hh signalling by directly activating the extracellular cysteine-rich domain in Smo [25]. Other Hh receptors that interact with Ptch and/or Smo have also been identified. The overexpression of Hh interacting protein (Hhip) attenuates Shh signalling activity, indicating that Hhip is a negative regulator of Hh signalling [26]. In addition, Hh ligands also bind to three known co-receptors: growth arrest-specific gene 1 (GAS1) [27], cell adhesion molecule-related, downregulated by oncogenes (CDO) [28], and Brother of CDO (BOC) [29, 30]. Activating these co-receptors promotes Hh-Ptch binding and enhances subsequent Hh signalling activity. Gli1, Gli2, and Gli3 processing and nuclear transport play a critical role in intracellular conduction in recipient cells [31]. In vertebrates, Gli2 and Gli3 (abbreviated as Gli2/3) can be categorised into three different forms: full-length (Gli2/3FL), repressor (Gli2/3 R), and activator (Gli2/3 A) [19]. Gli1 is a target of Hh signalling and acts as a transcriptional activator that amplifies the effects of Hh signalling [19, 32]. Gli2 is a principal transcriptional activator, while Gli3 has dual functions of transcriptional activation and repression [19, 32]. Sufu and kinesin family member 7 (Kif7) are two core regulators of mammalian Gli proteins [33]. Deletion of Sufu in mice results in strong activation of Hh signalling, suggesting that Sufu is a negative regulator of Shh signalling [34]. Kif7 moves from the basal body to the tip of the PC in an Hh-dependent manner, assisting Smo accumulation in the PC [35]. Multiple protein kinases, including protein kinase A (PKA), glycogen synthase kinase 3β (GSK3β), and casein kinase 1α (CK1) regulate the phosphorylation of Gli family proteins and are associated with the basal body of the PC [36].

**Fig. 1:**
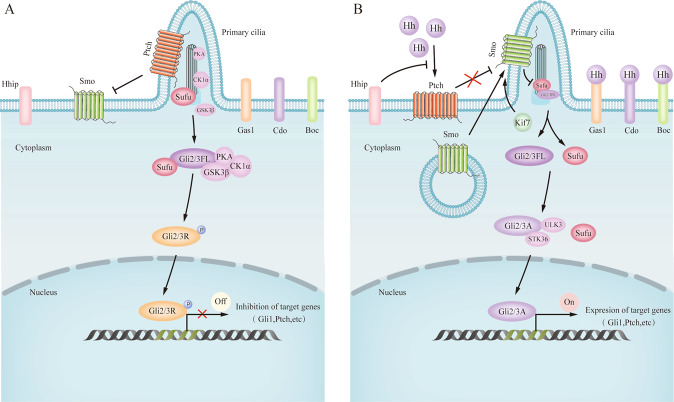
Schematic diagram of paracrine Hh signalling in the mammalian intestine. Hh ligands are secreted by intestinal epithelial cells. The signals are received by various types of mesenchymal cells. In the absence of Hh ligands (**A**), the receptor Ptch localises in the primary cilium (PC) and inhibits Smo, a necessary receptor to deliver Hh signals, movement to the primary PC. Under such cases, Sufu restrains the full-length form of Glioma-associated oncogenes 2/3 (Gli2/3FL) in the cytosol, where they are partially phosphorylated and proteolyzed by a multiple protein kinase complex composed of PKA, GSK3β, and CK1α. This generates the repressor form, namely Gli2/3 repressor (Gli2/3 R), which translocates to the nucleus to bind to and inhibit the expression of Hh target genes. In the presence of Hh ligands (**B**), binding of Hh ligands to Ptch removes the inhibitory action of Ptch on Smo and drives the accumulation and activation of Smo in the PC, leading to subsequent inhibition of Sufu by Smo and the dissociation of Gli2/3FL. kinesin family member 7 (Kif7), a core regulator of mammalian Gli proteins, moves from the basal body to the tip of the PC in a Hh-dependent manner, assisting Smo accumulation in the PC. Gil2/3FL is then phosphorylated by serine/threonine protein kinases, including ULK3 and STK36, resulting in the formation of Gli2/3 activator (Gle2/3 A) and translocation into the nucleus. Once in the nucleus, Gli2/3 A upregulates the transcriptional targets of Hh signalling, including Ptch and another Hh-binding protein, Hhip. Gas1, Cdo, and Boc are co-receptors that promote Hh-Ptch binding. Arrows and blunt ends lines indicate activation and inhibition, respectively. Ptch Patched, Smo Smoothened, Hhip Hedgehog interacting protein, Gas1 Growth arrest-specific gene 1, Cdo Cell adhesion molecule-related, downregulated by oncogenes; Boc, Brother of Cdo; Gli2/3FL, full-length form of Glioma-associated oncogenes 2/3, Gli2/3 A activator form of Glioma-associated oncogenes, Gli2/3 R repressor form of Glioma-associated oncogenes 2/3, Sufu Suppressor of Fused, Kif7 kinesin family member 7, PKA protein kinase A, GSK3β glycogen synthase kinase 3β, CK1α casein kinase 1α STK36 serine/threonine protein kinase 36, ULK3 UNC-51-like kinase 3.

In the absence of Hh ligands, Ptch localises within the PC and inhibits Smo movement to the PC. In this case, Sufu restricts the full-length form of Gli2/3FL to the cytosol, where it is partially phosphorylated and proteolyzed by a multiple protein kinase complex composed of PKA, GSK3β, and CK1α to form its repressor form, namely Gli2/3 R. Then, Gli2/3 R translocates to the nucleus to bind to and inhibit the expression of Hh target genes [35]. In the presence of Hh ligands, Smo activation inhibits Sufu in the PC [14], which allows Gli2/3FL to dissociate from Sufu and be phosphorylated by UNC-51-like kinase 3 (ULK3) and serine/threonine protein kinase 36 (STK36) to form Gli2/3 A. Then, GLi2/3 A moves to the nucleus to enhance the expression of Hh target genes, such as *Gli1* and *Ptch* [14] (Fig. 1).

### Noncanonical Hh signalling

Noncanonical Hh signalling currently refers to Hh co-receptor-dependent signalling without the assistance of the canonical Hh-Ptch-Smo-Gli pathway [35, 37]. It can be categorised into three distinct subtypes: (1) Ptch-mediated noncanonical Hh signalling, which works through Ptch1 but does not require Smo; (2) Smo-dependent/Gli-independent noncanonical Hh signalling, which operates through Smo functions without the regulation of Gli; and (3) Smo-independent/Gli dependent noncanonical Hh signalling [38]. The noncanonical Hh pathway plays a critical role in various diseases, such as colon cancer [39] and cholangiocarcinoma [40]. However, it remains unclear whether noncanonical Hh signalling is implicated in the pathogenesis of IBD.

### Feedback in the Hh pathway

Hhip is a negative regulator of Hh signalling and inhibits Hh-Ptch signalling [26]. In addition, Sufu is inhibited by Smo in PC [34]. In the Hh pathway, there are many positive regulators, such as GAS1, CDO, and BOC, which are transcriptionally inhibited by Hh stimulation, resulting in feedback that restrains Hh pathway activity [27, 30]. In addition, *Gli1* [11, 19] and *Gli2* [41, 42] appear to function as a positive feedback provider, thus amplifying the effects of Hh signalling. These feedback loops contribute to the maintenance of homeostasis in the Hh signalling pathway.

## Physiological role of Hh signalling in the gut

Shh and Ihh ligands are expressed in the intestine [43] and are highly expressed in the endoderm of the intestine before villus formation in the embryo [44]. As the villi in the small intestine develop, Ihh and Shh ligands are distributed to the villous base [44, 45]. In the colon, Ihh ligands are expressed in epithelial cells, while Shh ligands are confined to crypt cells [44, 45]. The expression of Shh and Ihh ligands is significantly reduced in the adult intestinal tract [44]. The Hh pathway is mainly paracrine in the intestine, where Hh ligands are secreted by epithelial cells and received by underlying mesenchymal cells [46]. Specifically, Ptch1 is expressed in the lamina propria of the small intestine and colon [47]. Herein, we discuss the important roles of Hh signalling in the formation of the intestinal structure, intestinal epithelial homeostasis, and inflammation in the intestinal tract.

### Hh signalling in the formation of intestinal structure

Hh signalling is critical in mammalian intestinal organogenesis and plays an important role in intestinal structures, particularly during villus formation [45].

The size of the villi is significantly reduced in Ihh-deficient mice [45]. Haramis et al. [48] found that bone morphogenetic protein 4 (BMP4) is only expressed in the mesenchyme. In villus-Noggin (a BMP inhibitor)-transgenic mice, heterotopic crypt structures were found on the villus, very similar to the phenotype observed in epithelial-Hhip-transgenic mice [48], suggesting cooperation between BMP and Hh signalling in crypt-villus axis formation. Notably, forkhead transcription factors (Foxf) are expressed in intestinal fibroblasts and are target genes of Hh signalling [49, 50]. Ormestad et al. [49] demonstrated that Foxf proteins function as mesenchymal factors that link Hh to BMP and Wnt signalling, thus regulating epithelial cell proliferation and survival. Walton et al. [51] showed that Hh signals regulate the initial formation of each villus by controlling the aggregation of mesenchymal clusters, which clusters express Ptch1 and Gli1. Specifically, enhanced Hh signalling promotes cluster formation and villus development, whereas inhibition of Hh signalling hinders villus emergence where clusters are not yet formed. Moreover, Hh signalling regulates mouse mesenchymal cell aggregation, which is required for epithelial remodelling during villus formation through the activation of nonclassical cadherin, thus providing a new mechanism for villi formation [52].

### Hh signalling in intestinal epithelial homeostasis

Intestinal epithelial homeostasis is characterised by rapid and constant epithelial regeneration that requires a strictly controlled balance between intestinal stem cell (ISC) proliferation and differentiation [53]. Importantly, the Hh signalling pathway in the crypt-villus axis of the mammalian intestine maintains ISC homeostasis [54]. Ihh-knockout mice show a significantly reduced number of villi and reduced cell proliferation in the stem cell compartment [45], suggesting that Ihh is essential for ISC maintenance. Additionally, Kosinski et al. [55] demonstrated that intestinal epithelial Ihh regulates ISC regeneration and differentiation by signalling to the mesenchymal compartment and controlling the generation and proliferation of mesenchymal cells. Specifically, the loss of intestinal epithelial Ihh destroys the intestinal mesenchymal structure, disturbs crypt polarity and structure, hinders enterocyte differentiation, and increases the ectopic ISC proliferation, which is accompanied by an increase in epithelial Wnt signalling. Importantly, Degirmenci et al. [56] showed that subepithelial mesenchymal Gli1-expressing cells comprise the essential Wnt-producing stem cell niche in the colon, where they serve as a reserve Wnt source to promote ISC renewal and maintain intestinal epithelial homeostasis.

### Hh signalling in the regulation of intestinal inflammation and immunity

Hh signalling is involved in the regulation of intestinal inflammation and immunity. Lees et al. [57] provided the first evidence that Hh/Gli1 signalling is required for proper regulation of inflammatory responses in the mammalian gut, especially in IBD. Another study using a bi-transgenic mouse model of chronic Hh inhibition showed that persistent blockade of Hh signalling causes crypt hyperplasia and villus loss, leading to the development of intestinal inflammation [58]. In addition, Lee et al. [59] reported that genetic or pharmacological inhibition of the Hh pathway reduces colitis in mice by regulating stromal IL-10 expression and the number of CD4^+^Foxp3^+^ regulatory T cells. Notably, previous studies showed that there is a close relationship between the Hh signalling and tumour necrosis factor-α (TNF-α)/nuclear factor-kappa B (NF-κB), which deserves further attention. Specifically, the histone demethylase, jumonji domain-containing protein 2D (JMJD2D), which is induced by TNF-α/NF-κB signalling, can act as an upstream signal of Hh to induce increased Hh signalling, thereby protecting mice from dextran sodium sulphate (DSS)-induced colitis [60]. In addition, Shh was found to be a target gene of NF-κB, and the NF-kB/Shh axis promotes apoptosis resistance [61].

Interestingly, a recent study found that Ihh is involved in the healing of intestinal wounds and that Ihh deficiency activates multiple aspects of intestinal wound healing via BMP and activin signalling [62]. Elevated levels of Shh signalling have been detected in inflammatory gut diseases, suggesting that Shh is involved in epithelial healing [47]. In addition, in the fluorouracil (5-FU)-induced intestinal injury model, Shh signalling was decreased during the injury stage, whereas it was upregulated in the subsequent repair stage [43].

Thus, understanding how Hh signalling is implicated in intestinal inflammation and immunity is crucial for future studies on the regulation of Hh signalling in IBD, since IBD is a chronic and recurrent intestinal inflammatory disease.

## Crosstalk between Hh and other signalling pathways

The Hh signalling pathway interacts with multiple signalling pathways, especially Wnt and NF-κB signalling, which regulate mammalian intestinal homeostasis and play critical roles in IBD [63–65].

**Fig. 2:**
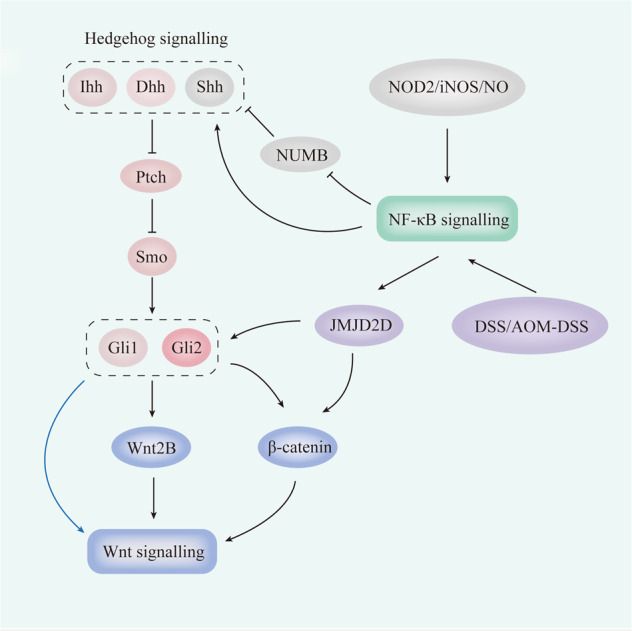
Crosstalk between Hedgehog and other pathways in the intestinal tract. β-catenin is a target gene of Hh/Gli2 signalling. Hh signalling can enhance Wnt signalling by inducing the expression of β-catenin in the colon during IBD and CAC. Epithelial Ihh and Shh upregulate the expression of Wnt2B in intestinal mesenchymal cells during chronic intestinal inflammation. The subepithelial Gli1-positive mesenchymal cells express high levels of Wnt2B. Dhh-activated Hh signalling positively regulates epithelial cell-produced Wnt2B expression following IEC injury and regeneration. Gli1-expressing mesenchymal cells serve as a reserve Wnt source during recovery from DSS-induced colitis in mice, thereby enhancing Wnt signalling. Shh is a target gene of NK-κB and can be induced by the activation of NK-κB in inflamed intestinal epithelial cells. NK-κB mediates NOD2-iNOS/NO-NUMB-mediated Shh signalling activation, which plays a role in modulating inflammatory responses in IBD. TNF-a/NF-κB signalling promotes the activation of Hh/Gli1/Gli2 signalling in DSS- or AOM/DSS-induced colitis. Arrows and blunt ends indicate activation and inhibition, respectively. The blue arrows indicate a special condition where Gli1 is overexpressed in mesenchymal cells. NOD2 the nucleotide-binding oligomerization domain 2, iNOS inducible nitric-oxide synthase, NO nitric oxide, NF-κB nuclear factor-kappaB, Ihh Indian hedgehog, Dhh Desert hedgehog, Shh Sonic hedgehog, Ptch Patched, Smo Smoothened, Gli1 Glioma-associated oncogene 1, Gli2 Glioma-associated oncogene 2, AOM-DSS azoxymethane and dextran sodium sulphate, JMJD2D jumonji domain-containing protein 2D.

Wnt-β-catenin signalling plays a key role in crypt-villus axis patterning and ISC homeostasis [66–69]. Wnt ligands are generated by intestinal Paneth cells surrounding leucine-rich repeat-containing G protein-coupled receptor 5^+^ (Lgr5^+^) ISCs [70] and mesenchymal cells [71, 72]. Blockade of Wnt secretion leads to a reduction in intestinal epithelial cell (IEC) proliferation and disruption of epithelial homeostasis, whereas exogenous Wnt ligands contribute to intestinal homeostasis by re-establishing Wnt/β-catenin signalling [68, 69]. Further, Wnt-β-catenin signalling is highly expressed in the inflamed mucosa of IBD patients [6] and is targeted in IBD treatment [73]. Importantly, previous literature shows that β-catenin is a target gene of Hh/Gli2 signalling and that Hh signalling can enhance Wnt signalling by inducing the expression of β-catenin in the colon of IBD and CAC patients [60]. Wnt2B is overexpressed in IBD and is a key extra-epithelial Wnt ligand that can induce Wnt/β-catenin signalling and maintain intestinal homeostasis [64]. Epithelial Ihh and Shh upregulate expression of Wnt2B in intestinal mesenchymal cells during chronic intestinal inflammation [74]. Consistently, subepithelial Gli1-positive mesenchymal cells express high levels of Wnt2B [69]. In addition, Dhh-activated Hh signalling positively regulates epithelial cell-produced Wnt2B expression following IEC injury and regeneration [75]. Recently, Degirmenci et al. [56] demonstrated that Gli1-expressing mesenchymal cells comprise the Wnt-producing stem cell niche and serve as a reserve Wnt source during recovery from DSS-induced colitis in mice (Fig. 2).

The NF-κB protein family regulates immune and inflammatory responses [76]. The canonical NF-κB pathway can be mediated via the TNF-α receptor and is stimulated by TNF-α [77]. NF-κB, whose activation is dramatically enhanced in IBD patients, is critical for the regulation of mucosal inflammation in IBD [63]. Importantly, Kasperczyk et al. [61] identified that Shh is a target gene of NK-κB and can be induced by NK-κB activation in inflamed intestinal epithelial cells [74]. NK-κB also mediates the nucleotide-binding oligomerization domain 2 (NOD2)-inducible nitric-oxide synthase (iNOS)/nitric oxide (NO)-NUMB endocytic adaptor protein (NUMB)-mediated Shh signalling, which plays a role in modulating inflammatory responses in IBD [78]. Recently, Zhuo et al. [60] demonstrated that TNF-α/NF-κB/JMJD2D signalling protects against DSS- or azoxymethane and DSS (AOM-DSS)/DSS-induced colitis via activation of Hh/Gli1/Gli2 and Wnt/β-catenin signalling. Mechanistically, DSS or AOM/DSS administration impairs IECs in the mouse colon and results in subsequent enteric bacterial translocation, leading to the stimulation of TNF-a/NF-κB and the induction of JMJD2D in the colonic epithelium. This, in turn, leads to the activation of Hh/Gli and Wnt/β-catenin signalling, thus protecting against colitis-induced apoptosis and promoting proliferation (Fig. 2).

Taken together, an in-depth understanding of the crosstalk between the Hh and other signalling pathways will help us to better understand the role of Hh in IBD.

## Hh and IBD

Hh signalling plays a key role in homeostasis, cell death, and cytokine stimulation in the intestinal epithelium. Significantly, Hh signalling is involved in the pathogenesis of IBD and CAC.

### The role of Hh signalling in IBD

Hh signalling in the inflamed mucosa of IBD patients is lower than that in normal mucosa or tissues [6]. Blockade of Hh signalling worsens IBD [6]. Lees et al. [57] revealed that Hh signalling activity is decreased in inflammatory mucosal colonic areas of IBD patients, especially in UC. In contrast, Buongusto et al. [6] showed reduced expression of Hh pathway components (Shh, Ihh, and Gli1) in the colonic mucosa of IBD patients, although the reduction was not as obvious as that in CD. This inconsistency may be attributed to genetic differences among the study populations. The Gli gene family was differentially expressed in normal left and right colons, while the expression of Gli in the sigmoid tissue of patients with inflammatory UC was downregulated by 1.5-fold compared with that in non-inflammatory UC tissue. This evidence further enhances the connection between the Gli gene and the pathogenesis of IBD [79].

Inhibition of Hh signalling also exacerbates IBD-associated colitis in mice, and can lead to intestinal inflammatory phenotypes such as villus atrophy and crypt hyperplasia [58, 59, 62]. Mice with a 50% reduction in Gli1 developed severe intestinal inflammation during DSS treatment compared with WT mice, suggesting that Gli1 protects mice from DSS-induced colitis [57]. Mechanistically, Hh/Gli1 signalling is necessary for the proper regulation of the intestinal response to the acute inflammatory challenge by targeting local myeloid cells and upregulating the expression of cytokines including interleukin (IL)-12, IL-17, and IL-23 [57]. In addition, Hh signalling is activated by histone demethylase JMJD2D during DSS-induced colitis in mice, leading to the amelioration of colitis [60]. Loss of intestinal epithelial Ihh or a lack of Smo in Hh target cells made the animals more sensitive to DSS-induced colitis, while activation of Ihh sequesters C-X-C motif chemokine ligand 12 (CXCL12) in fibroblasts, thus damaging immune cell migration and inhibiting the immune response [80]. Moreover, Hh signalling decreases inflammatory cytokine levels, including TNF-α, IL-17, and transforming growth factor (TGF)-β, which reduces monocyte chemoattraction and fibroblast proliferation but promotes fibroblast migration. Thus, Hh signalling is strongly involved in intestinal inflammation and may be a new target for IBD treatment [6]. Significantly, genetically or pharmacologically activating Hh signalling has been reported to ameliorate colitis by inducing the expression of IL-10 in Hh-responsive stromal cells, which subsequently increases the amount of CD4^+^Foxp3^+^ regulatory T cells [59].

Genetic factors may be involved in the pathogenesis of IBD. Previous genetic linkage studies identified a critical contribution of the IBD2 locus, localised at 12q13, to IBD susceptibility [81, 82]. Of note, Gli1, a key transduction factor of the Hh signalling pathway, is a strong candidate gene that maps to the IBD2 locus, an IBD-susceptibility linkage region [57]. Decreased function of the transcription factor Gli is involved in the pathogenesis of IBD [57]. Polymorphisms in the NOD2 gene also play a significant role in IBD aetiology. Ghorpade et al. [78] illustrated that the activation of Shh signalling is facilitated by NOD2-driven inflammation in a murine model of IBD. Further, Shh signalling is beneficial for the remission of intestinal inflammation, which is mediated by NO-responsive microRNA (miR)-146a. Crosstalk between NOD2 and Shh signalling contributes to the elevated expression of inflammatory genes, including IL-12, TNF-α, IL-6, CCL-5, and CXCL-9, and thus to the regulation of gut inflammation [78].

### The role of Hh signalling in CAC

Patients with IBD have an increased risk of gastrointestinal malignancies. CAC is among the most frequently occurring cancers in IBD patients [83]. Chronic inflammation contributes to CAC by enhancing cell proliferation and inducing genetic mutations in tumour suppressors [84].

**Fig. 3:**
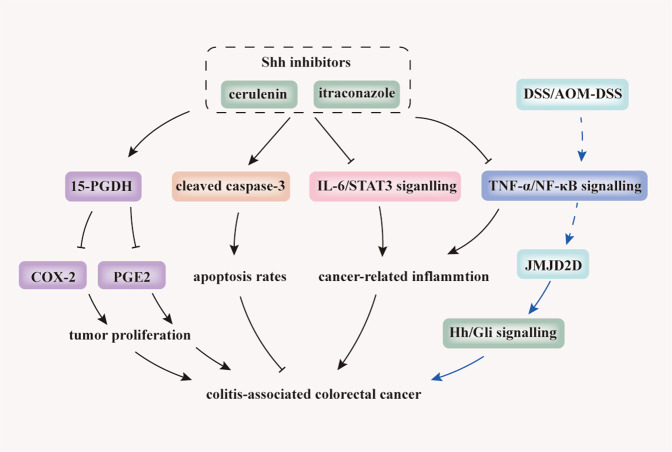
Inhibition of Hh signalling prevents tumourigenesis and CAC progression. Shh inhibitors, including cerulenin and itraconazole, play a potent suppressing role on carcinogenesis-associated inflammation by downregulating IL-6, IL-6-associated signal transducer and activator of transcription 3 (STAT3) signalling, and NF-κB-related TNF-α. Moreover, Shh inhibitors induce apoptosis by elevating cleaved caspase-3 and exert an anti-proliferating effect by elevating 15-hydroxyprostaglandin dehydrogenase (15-PGDH), a prostaglandin E2 (PGE2)-degrading and tumour-suppressive enzyme. This subsequently inhibits cyclooxygenase-2 (COX-2). In addition, activation of Hh signalling by TNF-α/NF-κB-generated overexpression of the histone demethylase, JMJD2D, can be impaired by DSS or AOM/DSS and promotes tumourigenesis and CAC metastasis. Blue lines indicate evidence from two separate studies. Arrows, blunt ends, and dotted lines indicate activation, inhibition, and indirect regulatory effects, respectively. 15-PGDH 15-hydroxyprostaglandin dehydrogenase, PGE2 prostaglandin E2, COX-2 cyclooxygenase-2; IL-6 interleukin-6, STAT3 signal transducer and activator of transcription 3, JMJD2D jumonji domain-containing protein 2D, TNF-α/NF-κB Tumour necrosis factor-α/nuclear factor-kappaB.

The most widely used model for studying CAC is AOM-DSS-induced colitis [85]. Given that Hh/Gli1 signalling is dysregulated in human colon cancer cells and is a critical factor for tumour progression and metastasis [86], we next discuss the role of Hh signalling in CAC. Kangwan et al. [87] demonstrated that Shh inhibitors, including cerulenin and itraconazole, play potent suppressing roles in carcinogenesis-associated inflammation by downregulating IL-6, IL-6-associated signal transducer and activator of transcription 3 (STAT3) signalling, and NF-κB-related TNF-α. Moreover, Shh inhibitors induce apoptosis by increasing the levels of cleaved caspase 3. Shh inhibitors also exert an anti-proliferative function by elevating 15-hydroxyprostaglandin dehydrogenase (15-PGDH), a prostaglandin E2 (PGE2)-degrading and tumour-suppressive enzyme [88], which subsequently inhibits cyclooxygenase-2 (COX-2) (Fig. 3). Zhuo et al. [60] illustrated that the activation of Hh signalling by TNF-α/NF-κB-generated overexpression of JMJD2D, which is impaired by DSS or AOM/DSS, promotes tumorigenesis and metastasis in CAC (Fig. 3).

**Fig. 4:**
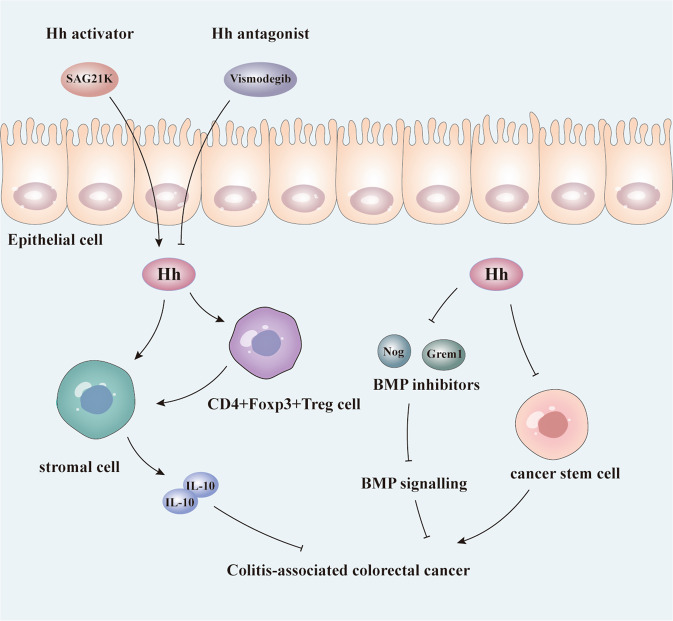
Activation of stromal Hh signalling reduces tumour burden and CAC progression. Activation of stromal Hh signalling inhibits the initiation and progression of CAC by increasing stromal anti-inflammatory cytokine IL-10 and CD4 + Foxp3+regulatory T cells. The Hh activator SAG21k can decrease colitis severity and CAC burden. Administration of the Hh antagonist, vismodegib, can facilitate CAC formation. Additionally, activated stromal Hh signalling reduces the tumour burden and tumour progression in mouse CAC models partly by reducing secreted BMP inhibitors such as Gremlin 1 (Grem1) and Noggin (Nog), and by restricting cancer stem cell (CSC) features. Arrows and blunt-ended lines indicate activation and inhibition, respectively. Hh Hedgehog, IL-10 interleukin-10, Nog Noggin, Grem1 Gremlin, BMP bone morphogenetic protein.

In contrast, activation of stromal Hh signalling was found to reduce tumour burden and progression in CAC. Lee et al. [59] demonstrated the oncogenic role of Hh inhibitors in CAC. Mechanistically, activation of stromal Hh signalling impairs the initiation and progression of CAC by increasing the levels of stromal IL-10 and CD4^+^Foxp3^+^ regulatory T cells. SAG21k, an Hh activator, can decrease colitis severity, and CAC burden. In contrast, the administration of vismodegib, an Hh antagonist, facilitates the formation of CAC (Fig. 4). This effect may be due to the tumour suppressing effect of stromal Hh, whereas its effects on colitis severity are irrelevant. Similarly, Gerling et al. [89] showed that activated stromal Hh signalling remarkably reduces tumour burden and tumour progression in mouse CAC models, partly by reducing the levels of secreted BMP inhibitors, such as Gremlin 1 (Grem1) and Noggin (Nog), and by reducing cancer stem cell (CSC) features (Fig. 4).

Together, these results suggest that the role of Hh signalling in CAC is complicated, and using Hh inhibitors to cure CAC may lead to IBD relapse [59]. Research on safe and potent Hh inhibitors/activators to attenuate colitis or prevent CAC progression need to be performed.

## Insights into the development of therapeutic strategies targeting the Hh pathway for IBD treatment

Hh agonists or antagonists may offer new treatment options for patients with IBD and may prevent the progression to CAC. While treating CAC, however, the risk of IBD recurrence should also be considered.

Hh treatment has been extensively studied in various cancers, including basal cell carcinoma [90, 91], medulloblastoma [92, 93], and pancreatic cancer [94, 95]. Vismodegib, a small molecule antagonist of Smo, has been approved by the FDA for the treatment of patients with advanced basal cell carcinoma [96, 97]. Another Smo antagonist, sonidegib, was used to treat patients with recurrent disease in locally advanced basal cell carcinoma or patients who did not comply with radiotherapy or surgical resection [98]. In addition to vismodegib and sonidegib, the Smo inhibitor glasdegib has been approved for the treatment of acute myeloid leukaemia (AML) in combination with low-dose cytarabine [99, 100]. Several Hh antagonists have been explored in other cancer types [101, 102]. Because of acquired cross-resistance to different types of Smo inhibitors and disappointing results in most clinical trials using Smo inhibitors to treat solid tumours, targeting Gli transcription factors may be a more effective approach to antitumour therapy [102, 103]. Patients with metastatic colon cancer treated with vismodegib were more likely to have adverse intestinal events, including diarrhoea, loss of appetite, and pain, than patients treated with placebos, which may suggest that Hh antagonists can damage the gut [104]. Notably, combining two Hh inhibitors that target Hh signalling at different layers, including low-dose aspirin (which resembles the JMJD2D inhibitor 5-c-8HQ) and vismodegib, can synergistically inhibit Hh signalling and CAC progression. This regimen may become a novel therapy [60]. Further research on the molecular mechanisms of Hh signalling and its role in the initiation and progression of IBD may lead to the development of new drugs such as Hh inhibitors or agonists for the treatment of IBD and CAC patients.

## Summary

Hh signalling plays a crucial role in embryogenesis and the maintenance of adult tissue homeostasis [36]. In the intestinal tract, Hh signalling is involved in intestinal structure, intestinal epithelial homeostasis, and inflammation. First, the Hh pathway is implicated in villus formation [45]. Second, Hh signalling in the crypt-villus axis regulates intestinal epithelial homeostasis by maintaining the balance between ISC proliferation and differentiation [54]. Third, Hh signalling is involved in the regulation of intestinal inflammation and immunity [58]. Hh/Gli1 signalling is required for the proper regulation of inflammatory responses in the mammalian gut, including IBD, which merits further investigation. Understanding how Hh signalling is implicated in intestinal inflammation and immunity will contribute to future studies on the regulation of Hh signalling in IBD pathogenesis.

Interestingly, several studies have uncovered the crosstalk between the Hh and other signalling pathways, including Wnt and NF-κB. β-catenin is a target gene of Hh/Gli2 signalling [60]. Hh signalling can enhance Wnt signalling by inducing the expression of β-catenin in the colon during IBD and CAC [60]. Gli1-expressing mesenchymal cells serve as a reserve Wnt source and modulate the Wnt-producing stem cell niche during recovery from DSS-induced colitis in mice [56]. Shh is a target gene of NK-κB and can be induced by the activation of NK-κB in inflamed intestinal epithelial cells [74]. Besides, TNF-α/NF-κB/JMJD2D has a protective effect against DSS- or AOM/DSS-induced colitis via activation of Hh/Gli1/Gli2 [60]. Understanding how Hh signalling interacts with these pathways will be helpful in elucidating the mechanisms whereby Hh signalling is implicated in the homeostasis of the intestinal epithelium and the pathogenesis of IBD.

Disruption of the Hh signalling pathway can lead to various diseases, including several cancers [105–107] and IBD [6, 57, 59, 60, 108]. Significantly, the expression of Hh signalling components in the inflamed mucosa of IBD patients is lower than that in normal mucosa or tissues, and blockade of Hh signalling worsens IBD [6]. Inhibiting Hh signalling can intensify IBD-associated colitis in mice, while genetically or pharmacologically activating Hh signalling ameliorates colitis [59]. Genetic factors have been suggested to be involved in the pathogenesis of IBD. In particular, IBD-susceptible genes, including IBD2 [81] and NOD2 [78], were reported to have a close relationship with Hh signalling. Gli1, the major transcription factor of Hh signalling, is a strong candidate gene that maps to the IBD2 locus, and can protect the intestines from damage from IBD-associated DSS-induced colitis [57]. Decreased Gli function is involved in IBD pathogenesis [57]. In addition, activation of Shh/Gli2 signalling is facilitated by NOD2-driven inflammation in a murine model of IBD and is beneficial for the remission of intestinal inflammation [78].

Of note, patients with IBD have a remarkable risk of gastrointestinal malignancies, among which CAC is one of the most frequently occurring cancers [84]. Current studies have shown the dual role of inhibiting Hh signalling in CAC. Some studies show that Hh inhibitors can prevent CAC [60, 87]. However, Hh inhibition has been shown to exacerbate CAC, while Hh agonists ameliorate IBD and CAC [59, 89]. Importantly, treating CAC with Hh inhibitors may lead to IBD relapse [59]. Therefore, while treating CAC, the risk of IBD recurrence should also be taken into consideration. A comprehensive understanding of the role of Hh signalling in CAC will help in the development and optimisation of therapeutic drugs that ameliorate or restrain CAC.

Hh treatment, including the Smo antagonists vismodegib [109], sonidegib [98], and glasdegib [110], has been approved for the treatment of patients with various diseases or cancers. Importantly, combining two Hh inhibitors that target Hh signalling at different layers, including aspirin or JMJD2D inhibitor 5-c-8HQ and vismodegib, can synergistically inhibit Hh signalling and CAC progression [60]. Therefore, Hh agonists or antagonists may offer new treatment options for patients with IBD and may prevent progression to CAC. These treatments warrant further clinical trials.

Collectively, the current therapeutic effects of current drugs for IBD treatment must be improved. Targeting components of Hh signalling via Hh agonists or antagonists may offer novel treatment options for patients with IBD and may prevent the progression to CAC. Further clinical research is warranted and may pave the way to decrease the global burden of IBD.

## Supplementary information

author contribution statement

## Data Availability

All data included in this review are available upon request by contact with the corresponding author.
