# Supplementary material for: Emerging roles of the Hedgehog signalling pathway in inflammatory bowel disease
Source: Cell Death Discov. 2021 Oct 26;7:314. doi: 10.1038/s41420-021-00679-7 (PMC8548344; doi:10.1038/s41420-021-00679-7)
Supplement: Supplementary file 1 — author contribution statement [file 41420_2021_679_MOESM1_ESM.pdf]

DECLARATION OF CONTRIBUTIONS TO ARTICLE

ADMC

Manuscript Number: CDDISCOERY-21-1856

Journal Name: *Cell Death Discovery* (the 'Journal')

Proposed Title of the Contribution: Emerging roles of the Hedgehog signalling pathway in inflammatory bowel disease (the 'Contribution)

Author(s):

Zhuo Xie, Mudan Zhang, Gaoshi Zhou, Lihui Lin, Jing Han, Ying Wang, Li Li, Yao He, Zhirong Zeng, Minhu Chen & Shenghong Zhang (the 'Authors')

For all *CDDiscovery* articles, each person named as an author in the published version must be able to show he or she has contributed substantially to the article.

Authorship credit should be based on 1) substantial contributions to conception and design, acquisition of data, or analysis and interpretation of data; 2) drafting the article or revising it critically for important intellectual content; and 3) final approval of the version to be published. Authors should meet conditions 1, 2 and 3.

Any person who cannot be shown to have made a substantial contribution to the article cannot be listed as an author in the final version. The name of any person who is deemed to have made a minor contribution can, however, appear in the Acknowledgments section of the article.

Please complete the table below to indicate the contributions of all named authors to the manuscript.

Author Full Name:	Specification of Contribution to the Manuscript:
Zhuo Xie	wrote and revised the manuscript，approved the final version of the manuscript
Mudan Zhang	wrote and revised the manuscript，approved the final version of the manuscript
Gaoshi Zhou	revised the contents of the manuscript，approved the final version of the manuscript
Lihui Lin	revised the contents of the manuscript，approved the final version of the manuscript
Jin Han	revised the contents of the manuscript，approved the final version of the manuscript
Ying Wang	revised the contents of the manuscript，approved the final version of the manuscript
Li Li	revised the contents of the manuscript，approved the final version of the manuscript
Yao He	revised the contents of the manuscript，approved the final version of the manuscript
Zhirong Zeng	revised the contents of the manuscript，approved the final version of the manuscript
Minhu Chen	revised the contents of the manuscript，approved the final version of the manuscript
Shenghong Zhang	Designed the study, revised the contents of the manuscript，approved the final version of the manuscript

Please complete the table below to indicate the contributions of all named authors to the figures.

Figure 1:

Mudan Zhang constructed the Figure 1, Zhuo Xie revised the Figure 1.

Figure 2:

Zhuo Xie and Mudan Zhang constructed and revised the Figure 2.

Figure 3:

Zhuo Xie constructed and revised the Figure 3.

Figure 4:

Zhuo Xie constructed and revised the Figure 4.

Figure 5:

Figure 6:

Signed for and on behalf of the Author(s):	Print Name:	Date:
	Shenghong Zhang	28 Sep, 2021
